# The Peroxisomal PTS1-Import Defect of *PEX1*- Deficient Cells Is Independent of Pexophagy in *Saccharomyces cerevisiae*

**DOI:** 10.3390/ijms21030867

**Published:** 2020-01-29

**Authors:** Thomas Mastalski, Rebecca Brinkmeier, Harald W. Platta

**Affiliations:** Biochemie Intrazellulärer Transportprozesse, Ruhr-Universität Bochum, Universitätsstr. 150, 44801 Bochum, Germany; thomas.mastalski@rub.de (T.M.); rebecca.brinkmeier@rub.de (R.B.)

**Keywords:** Atg36, Pex1, pexophagy, peroxisomal protein import

## Abstract

The important physiologic role of peroxisomes is shown by the occurrence of peroxisomal biogenesis disorders (PBDs) in humans. This spectrum of autosomal recessive metabolic disorders is characterized by defective peroxisome assembly and impaired peroxisomal functions. PBDs are caused by mutations in the peroxisomal biogenesis factors, which are required for the correct compartmentalization of peroxisomal matrix enzymes. Recent work from patient cells that contain the Pex1(G843D) point mutant suggested that the inhibition of the lysosome, and therefore the block of pexophagy, was beneficial for peroxisomal function. The resulting working model proposed that Pex1 may not be essential for matrix protein import at all, but rather for the prevention of pexophagy. Thus, the observed matrix protein import defect would not be caused by a lack of Pex1 activity, but rather by enhanced removal of peroxisomal membranes via pexophagy. In the present study, we can show that the specific block of *PEX1* deletion-induced pexophagy does not restore peroxisomal matrix protein import or the peroxisomal function in beta-oxidation in yeast. Therefore, we conclude that Pex1 is directly and essentially involved in peroxisomal matrix protein import, and that the *PEX1* deletion-induced pexophagy is not responsible for the defect in peroxisomal function. In order to point out the conserved mechanism, we discuss our findings in the context of the working models of peroxisomal biogenesis and pexophagy in yeasts and mammals.

## 1. Introduction

Peroxisomes are single membrane-bound organelles that are found in nearly all eukaryotic cells. Their most conserved function is the breakdown of fatty acids via beta-oxidation as well as the detoxification of the produced H_2_O_2_. While peroxisomes are specialized only on very long chain fatty acids (VLCFAs) in mammalian cells, they are the sole site for beta-oxidation of all fatty acids in yeast cells [1,2]. The important role of peroxisomes is highlighted by the occurrence of peroxisome biogenesis disorders (PBDs), which are severe human diseases that are caused by a reduction or loss of peroxisomal function [3,4].

The AAA (ATPases associated with diverse cellular activities)-type ATPases Pex1 and Pex6 and their membrane anchor Pex26 are essential peroxisomal biogenesis factors [5]. Their importance is indicated by the finding that 65% of all PBD patients suffer from mutations within the human AAA complex genes *PEX1* (48.5%), *PEX6* (13.1%) or *PEX26* (3.4%) [3]. Moreover, certain mutations in *PEX1*, *PEX6* or *PEX26* were recently shown to be the cause of the Heimler Syndrome [6,7].

The AAA complex has been linked to different cellular functions. The best established role concerns its requirement for peroxisomal matrix protein import [5]. Functional analysis in yeast and mammalian cells revealed that the AAA complex functions as dislocase for the ubiquitinated PTS1 (peroxisomal targeting signal type 1)-import receptor Pex5, enabling further rounds of PTS1-import [8,9,10].

Pex5 ferries the PTS1 cargo proteins from the cytosol to the peroxisomal docking complex and releases them into the peroxisomal matrix via a transient import pore. Finally, the monoubiquitination of Pex5 occurs on the conserved cysteine and primes Pex5 for the retrotranslocation by the AAA-type ATPase complex back to the cytosol. In case the export is impaired by a dysfunctional AAA complex, Pex5 gets polyubiquitinated on lysine residues and is degraded by the 26S proteasome. The occurrence and functional role of the different Ub-modifications of Pex5 are conserved from yeast to man. According to the published data from different organisms, the AAA-dependent removal of the unloaded Ub-Pex5 is thought to generate room for newly incoming cargo-bound Pex5 molecules, as the binding capacities at the peroxisomal membrane seem to be limited [8,9,10].

In case the entire peroxisome is destined for degradation, it is marked for the transport to the hydrolytic compartment of the cell—be it the vacuole in yeasts or the lysosome in mammals. While the basic mode of pexophagy is conserved, it is the recognition mechanism that displays species-specific differences. Mammalian peroxisomes exhibit ubiquitinated proteins that are recognized by ubiquitin-binding autophagy-receptors like Nbr1 or p62. Yeast peroxisomes contain peroxisome-specific adaptor proteins that act as pexophagy receptors, like Atg30 in *Pichia pastoris* or Atg36 in *Saccharomyces cerevisiae*. Both in mammalian and yeast cells, the corresponding membrane-bound receptor proteins link the organelle to the autophagy machinery via an interaction to Atg8/LC3 or Atg11. Subsequently, the formation of an autophagosomal membrane is initiated, which finally surrounds the organelle and transports it to the lysosomal compartment where it is degraded [11,12].

The observation has been described that the deletion of *PEX1* induces the constitutive degradation of peroxisomes in *S. cerevisiae* [13] and human cell culture [14]. Interestingly, the inhibition of the lysosome in human cells containing the Pex1(G843D) point mutant stopped the degradation, increased the number of peroxisomal structures and even partially elevated the overall beta-oxidation rate of VLCFAs in the cell [14]. Based on that study, a working model was published by another group [15], according to which the primary role of the mammalian AAA peroxins would be pexophagy prevention, and that they would only be indirectly linked to matrix protein import [15]. The model acknowledges that the AAA complex-mediated export of the ubiquitinated Pex5 is essential for the general peroxisomal function. However, the new idea is that Ub-Pex5 has to be removed by the AAA complex in order to eliminate the Ub-signal on peroxisomes. In case the AAA complex is impaired by a dysfunctional Pex1, the Ub-Pex5 would accumulate on the peroxisome, resulting in its recognition by Ub-binding autophagy receptors and the lysosomal degradation of the organelle. The matrix protein import defect in these cells is thought to occur because the target peroxisomal membranes are missing due to the fast degradation via pexophagy. According to this model, the block of pexophagy by inhibiting the lysosome stabilizes the peroxisomes and restores PTS1 protein import, even without fully functional Pex1, as its proposed function in pexophagy prevention has become redundant due to the lysosomal inhibitor [15]. This was described as a paradigm shift, as it suggested that the AAA complex per se would not be essential for matrix protein import. Moreover, it was suggested that the mentioned 65% of PBD cases with dysfunctional AAA complex constituents are caused by enhanced pexophagy rather than a primary defect in the matrix protein import mechanism [15].

In order to learn how Pex1 may link peroxisomal biogenesis and pexophagy, we wanted to test the claim that a genetic block of peroxisome degradation can complement the matrix protein import defect in *S. cerevisiae pex1*Δ cells.

## 2. Results

**Figure 1:**
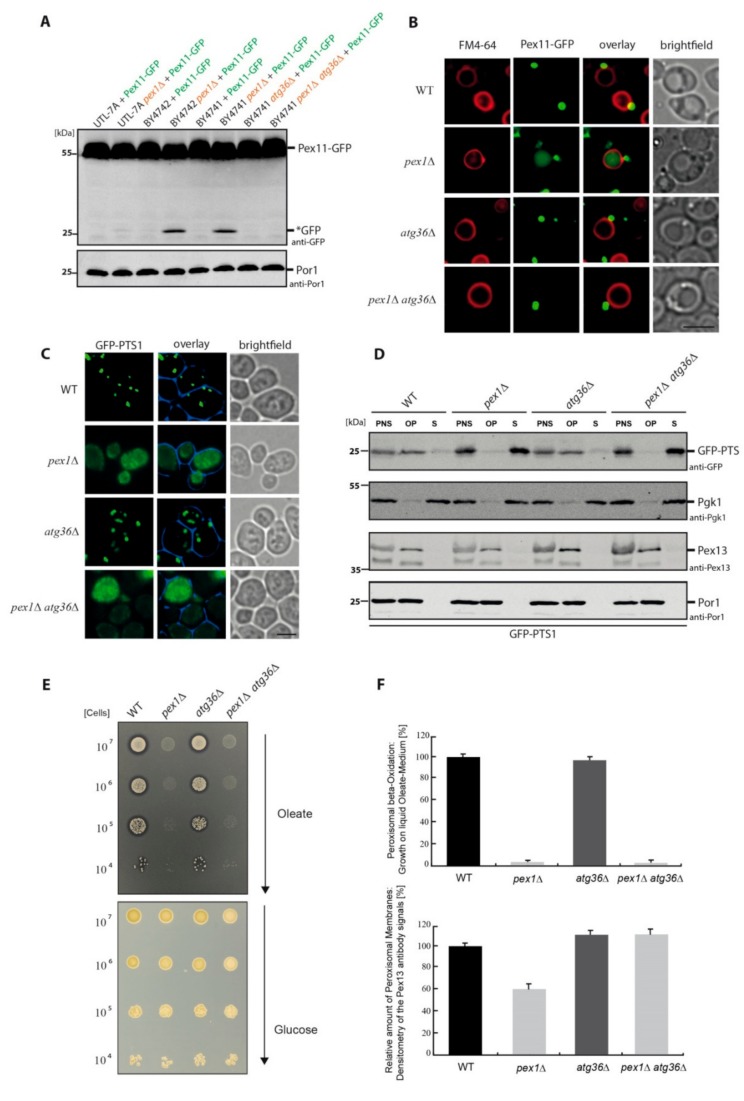
The matrix protein import defect in *PEX1*-deficient cells occurs independently of pexophagy. (**A**) The indicated different *Saccharomyces cerevisiae* wild-type (WT) strains were transformed with the peroxisomal membrane protein Pex11 genetically fused to green fluorescent protein (GFP). The autophagic degradation of peroxisomes is shown by the occurrence of free *GFP. The *pex1*Δ strains of the BY4741 and BY4742 background display a constitutive degradation of peroxisomes, while the UTL-7A *pex1*Δ strain lacks *PEX1* deletion-induced pexophagy. The degradation of peroxisomes depended on the presence of Atg36. The mitochondrial protein Por1 was used as loading control. Uncropped versions of the blots can be seen in the Supplemental Figure S1A. (**B**) The vacuolar membrane of BY4741 cells was stained red with FM4-64, while peroxisomal structures labeled with Pex11-GFP are visible as green dots. In addition, the *pex1*Δ strain displays a diffuse green staining within the vacuole, demonstrating that a portion of the peroxisome population was degraded via pexophagy. This degradation is fully blocked in the *pex1*Δ*atg36*Δ double mutant. Bar: 5µm. **(C)** Cells were transformed with a plasmid encoding the peroxisomal matrix protein marker GFP-PTS1. Cells with punctate pattern displayed a functional import, while cytosolic mislocalization indicated an import defect. Bar: 5µm. (**D**) The subcellular sedimentation analysis of the prepared post-nuclear supernatant (PNS) showed that the matrix protein GFP-PTS1 can mainly be detected in the organellar pellet (OP) fraction of WT and *atg36*Δ cells (intact import), while it is mislocalized to the cytosolic supernatant (S) fraction in *pex1*Δ and *pex1*Δ*atg36*Δ cells (import defect). The level of the endogenous peroxisomal membrane protein Pex13 was elevated in cells without Atg36. The mitochondrial Por1 and the cytosolic Pgk1 served as controls. Uncropped versions of the blots can be seen in the Supplemental Figure S1B. (**E**) The indicated strains were spotted as a series of 10-fold dilutions on a glucose medium as well as on a medium with oleate as the sole carbon source. The WT and *atg36*Δ strains had an intact peroxisome biogenesis and could grow on oleate plates. The utilization of oleate during beta-oxidation was further indicated by the formation of halos around the drop spots. The *pex1*Δ and *pex1*Δ*atg36*Δ strains were both unable to grow on oleate medium, indicating a defect in beta-oxidation and peroxisome function. (**F**) Lower diagram: The densitometry data from the Pex13-positive antibody signals from Western blots of organellar pellet fractions were compared. The block of pexophagy in *pex1*Δ*atg36*Δ resulted in a rise of the Pex13-level of the *pex1*Δ strain back to WT level. Upper diagram: The functionality of the indicated strains in beta-oxidation was analyzed by monitoring cell growth in liquid oleate medium (*n* = 3). The value for *PEX1-*deficient cells did not improve with the additional deletion of *ATG36*, suggesting that the block of pexophagy does not improve peroxisomal function in beta-oxidation.

The occurrence of *PEX1* deletion-induced pexophagy was tested in different genetic backgrounds of *Saccharomyces cerevisiae* strains (Figure 1A). The different wild-type (WT) strains and the corresponding *pex1*Δ deletion mutants were transformed with a plasmid encoding the peroxisomal membrane protein Pex11 genetically fused to green fluorescent protein (GFP). The autophagic degradation of peroxisomes is indicated by the occurrence of free *GFP in immunoblots, because GFP is relatively stable within the vacuole, while the Pex11-moiety of the fusion protein is degraded together with the rest of the organelle [16]. The *pex1*Δ strain of the BY4741 background displays a constitutive degradation of peroxisomes, as reported [13]. While the *pex1*Δ strain in the BY4742 background also exhibited a constitutive degradation, the UTL-7A *pex1*Δ strain nearly completely lacked *PEX1* deletion-induced pexophagy. We chose to continue with the BY4741 background and used a strain lacking the pexophagy receptor Atg36 [17].

We reassured that the *PEX1* deletion-induced constitutive degradation of peroxisomes was strictly dependent on the presence of Atg36 and that we were able to completely block this process in *pex1*Δ*atg36*Δ cells. As additional evidence, fluorescence microscopy was performed. The vacuolar membrane was stained with the red dye FM4-64, while peroxisomal membranes were labeled with Pex11-GFP (Figure 1B). The peroxisomes are visible as green dots. The *pex1*Δ strain displays puncta corresponding to peroxisomal structures. It also displays diffuse green staining within the vacuole, demonstrating that a part of the peroxisome population is constitutively degraded via pexophagy. This degradation is fully blocked in the *pex1*Δ*atg36*Δ double mutant because no green staining of the vacuolar lumen occurred.

It was important to elucidate whether Atg36 has an impact on the correct targeting and import of PTS1 matrix proteins. We were especially interested in the question of whether the block of pexophagy via the deletion of *ATG36* could partially restore the PTS1 import defect in *pex1*Δ cells. Therefore, we used fluorescence microscopy with the matrix marker protein GFP-PTS1 (Figure 1C). WT and *atg36*Δ cells showed a clear punctate pattern, which indicates a correct targeting and import of GFP-PTS1 in the peroxisomes of these strains. In contrast, the *pex1*Δ single mutant and the *pex1*Δ*atg36*Δ double mutant exhibited GFP-PTS1 that was mislocalized to the cytosol, indicating that both strains did not contain import-competent peroxisomal structures.

Because the cytosolic GFP signal could potentially cover the signal of a small GFP-PTS1 fraction that might possibly still have been imported into peroxisomal structures, the import efficiency of PTS1 cargo proteins was analyzed in more detail. To this end, the localization of GFP-PTS1 was monitored also via subcellular fractionation. Post-nuclear supernatants (PNS) were prepared from oleate-induced cells, which then were subjected to differential centrifugation (Figure 1D). The immunoblot data showed that the protein level of the endogenous peroxisomal membrane protein Pex13 was elevated in *pex1*Δ*atg36*Δ cells compared to that of *pex1*Δ cells. This indicates that the block of pexophagy stabilized peroxisomal structures and their membrane proteins, as also described for mammalian cells [14]. In the case of the PTS1 matrix protein import, however, the situation is different. The matrix protein marker GFP-PTS1 could mainly be detected in the peroxisome-containing organellar pellet (OP) fraction of WT and *atg36*Δ cells, indicating a functional import. In contrast, GFP-PTS1 clearly mislocalized to the cytosolic supernatant (S) fraction in *pex1*Δ and *pex1*Δ*atg36*Δ cells, demonstrating a clear import defect. Therefore, we found that the PTS1 import defect of *PEX1*-deficient cells was not restored when *ATG36* was deleted in addition. This result demonstrates that the peroxisomal import of GFP-PTS1 in *PEX1*-deficient cells remains inhibited in *pex1*Δ*atg36*Δ cells; therefore, this effect is independent of the block of pexophagy.

The basic remaining question was whether the peroxisomal function in beta-oxidation could be improved in *PEX1*-deficient cells with the block of pexophagy. Functional assays were performed by spotting a series of 10-fold dilutions of WT, *atg36*Δ, *pex1*Δ and *pex1*Δ*atg36*Δ cells on glucose medium plates as well as on plates with a medium containing oleic acid as the sole carbon source (Figure 1E). Because peroxisomes are the only site in yeast cells that can utilize fatty acids via beta-oxidation, peroxisomes become essential for viability under these conditions. The WT and *atg36*Δ strain grew on oleate plates and, therefore, displayed an intact peroxisome biogenesis. The utilization of oleate during beta-oxidation was further indicated by the formation of halos around the drop spots where oleate was consumed. The *pex1*Δ and *pex1*Δ*atg36*Δ strains were both unable to grow on oleate medium. Similarly, no halos were formed, indicating a defect in beta-oxidation in both cases.

Finally, the effect of pexophagy inhibition on the stabilization of peroxisomal membrane structures was correlated with the efficiency of peroxisomal function in beta-oxidation (Figure 1F). We used organellar pellet samples (as in Figure 1D) and compared the densitometric data from the antibody signals for the peroxisomal membrane protein Pex13 as an indicator for the relative amount of peroxisomal membrane structures in the corresponding strains (Figure 1F, lower diagram). The values were normalized using the mitochondrial membrane protein Por1 as loading control. In line with our observation (Figure 1D), the number of Pex13-positive signals was significantly diminished in *pex1*Δ cells compared to WT. The additional block of pexophagy in the *pex1*Δ*atg36*Δ strain seemed to help to significantly stabilize peroxisomal membrane structures when compared to *pex1*Δ cells because the Pex13-positive signals reached, again, a level comparable to WT and *atg36*Δ cells.

Next, the functional activity of peroxisomes of the same strains was analyzed by monitoring cell growth in liquid oleate medium as relative growth efficiency compared to WT cells (Figure 1F, upper diagram). This method is relatively sensitive and should be able to detect minor changes in growth dynamics [18]. However, the value for *pex1*Δ cells did not improve with the additional deletion of *ATG36*. The *pex1*Δ and *pex1*Δ*atg36*Δ strains showed no statistically significant differences, as both exhibited no real growth in liquid oleate medium. This finding strongly suggests that the block of *PEX1* deletion-induced pexophagy via the additional deletion of the pexophagy receptor gene *ATG36* does not improve peroxisomal function in beta-oxidation.

In summary, the results show that, although the block of pexophagy in a *pex1*Δ*atg36*Δ strain does result in stabilization of Pex13-positive membranes compared to the *pex1*Δ strain, this effect does not correlate positively with the peroxisomal function in beta-oxidation, which is not improved. The increase in Pex13-containing peroxisomal membrane structures within pexophagy-deficient cells did not result in an increase in peroxisomal beta-oxidation activity. Therefore, the PTS1 import defect of a *pex1*Δ strain is independent of *PEX1* deletion-induced pexophagy and is not rescued by the inhibition of pexophagy via the deletion of *ATG36*.

## 3. Discussion

In the present study the question was asked whether the matrix protein import defect and functional impairment of a *PEX1*-deficient strain could be rescued by a specific block of pexophagy via the additional deletion of *ATG36* in *S. cerevisiae*.

This question is of relevance because it is important for the understanding of the general role of Pex1 in peroxisome function and homeostasis and the resulting working model. Based on data of a recent study with mammalian cells [14], a model was proposed that suggests an indirect role of Pex1 in matrix protein import [15]. This was mainly based on the finding that the chemical inhibition of the lysosome, and therefore also of pexophagy, partially restored peroxisomal function on the cellular level in the case of the Pex1(G843D) point mutant [14]. Pex1(G843D) is the most common *PEX1* mutation in PBD patients and can be found in approximately 25% of all cases [19]. It displays a rather mild clinical phenotype, which might be caused by a partial misfolding of the protein [20,21]. Therefore, it has been demonstrated before that Pex1(G843D) cells can recover Pex1(G843D)-, Pex6- and Pex5-protein levels when they are treated with chaperone-like small molecules or by lowering the incubation temperature [21,22,23]. However, because of the residual activity of the point mutant Pex1(G843D), which has been estimated to achieve ca. 15% complementation activity [19], it is important to test also the *PEX1*-deletion cells that completely lack *PEX1* in order to fully understand the contribution of the AAA-type ATPase Pex1.

We tested *PEX1* deletion-induced pexophagy in the model organism *S. cerevisiae* and detected it in the BY4741 [13] and the BY4742 genetic background, but interestingly not in the UTL-7A background, which might be a future tool to find relevant factors involved in this process.

However, although we were able to specifically block *PEX1* deletion-induced pexophagy and to accumulate Pex13-positive peroxisomal membranes in BY4741 via the additional deletion of *ATG36*, we found in all our experiments concerning PTS1 matrix protein import and peroxisomal function in beta-oxidation that the *pex1*Δ*pex36*Δ strain behaved always similar to the *pex1*Δ strain. Both displayed cytosolic mislocalization of GFP-PTS1 in the fluorescence microscope, showing no change in the subcellular distribution of GFP-PTS1 in fractionation assays and exhibiting no growth on oleate plates or liquid oleate-medium. Therefore, we can conclude that the specific block of *PEX1* deletion-induced pexophagy neither restores PTS1 matrix protein import nor beta-oxidation; therefore, pexophagy is not responsible for the functional defects in a *pex1*Δ strain in *S. cerevisiae*.

The first published working model concerning the weighting of the roles of Pex1 in protein import and pexophagy still distinguished between mammalian and yeast cells [15]. The proposed idea was, as described above, that mammalian Pex1 would be mainly required for pexophagy prevention and only indirectly for peroxisomal PTS1 matrix protein import, while yeast Pex1 would be primarily required for PTS1 import and only secondarily needed for pexophagy prevention [15]. The latter assumption was based on the observation that the induced depletion of Pex1 via a degron-tag seemed to inhibit PTS1 import faster than it induced pexophagy, as shown in separate experiments via the fluorescence signals of GFP-PTS1 or Pex11-GFP, respectively [13]. In our study, we aimed to exclude the possibility that different dynamics in the underlying signaling and transport pathways might be altered in separate experiments; therefore, we chose to inhibit pexophagy specifically and completely by the deletion of *ATG36* in *pex1*Δ cells. We could clearly demonstrate that the block of *PEX1* deletion-induced pexophagy did not recover PTS1 matrix protein import and peroxisomal function.

Moreover, it is important to note the Law et al. study [14] itself provides evidence that supports our findings in yeast. The study utilized not only the mentioned point mutant but, in some experiments, also the deletion mutant (called *PEX1* null). Functional assays carried out upon chemical pexophagy inhibition demonstrated that cells with Pex1(G843D) partially recovered enough activity to breakdown significant amounts of VLCFAs, while the cells completely lacking Pex1 did not recover beta-oxidation to this extent [14]. This could be explained by the fact that the Pex1(G843D) point mutant is still partially active in matrix protein import, as principally shown before [19]. The block of pexophagy stabilizes these partially active Pex1(G843D)-containing peroxisomal structures and accumulates enough activity to breakdown a significant portion of VLCFAs. Indeed, this effect is not detected in cells that completely lack *PEX1* and matrix protein import [14]. The data show that residual biochemical activity of the AAA complex has to be present in order to allow the partial recovery of physiologic function to occur in cells with blocked pexophagy.

This fundamental distinction between the data from Pex1(G843D) and *PEX1*-deleted cells was not evident in the model [15] and was not further considered in several subsequently published reviews by other groups that cited the study and the model, resulting in the not accurate general impression that the entire AAA complex, per se, would not directly be required for the mechanism of matrix protein import at all.

This point might have been originally based on the circumstance that, on the clinical level, both the Pex1(G843D) mutant as well as a potential complete deletion of *PEX1* would lead to PBD in patients. However, on the biochemical level, Pex1(G843D) still has residual activity and enables at least a partial protein import. This represents a different biochemical situation compared with a complete loss of matrix protein import in *PEX1*-deleted cells. Therefore, it is the residual biochemical activity of Pex1(G843D) in matrix protein import that makes the beneficial effects of pexophagy inhibition, as described by Law et al. [14], in these patient cells possible.

Although thinkable, it might be too early to say that pexophagy is responsible for 65% of all cases of PBDs, as proposed by [15] and then cited by other reviews. Based on the data of the Law et al. study [14], it can be said that pexophagy is responsible for the drastic worsening of the pathophysiological phenotype of approximately 25% of PBD cases. This is the percentage of cases involving the Pex1(G843D) mutation [19] and is still a significant number. For the other Pex1-, Pex6- and Pex26-mutations found in PBD patients, it will have to be tested individually whether they still allow the occurrence of peroxisomal structures harboring sufficient residual AAA activity in matrix protein import, which then could accumulate upon lysosome inhibition to ensure a certain import rate of beta-oxidation enzymes and therefore a basal physiologic functionality. Therefore, this concept has also implications on the possible future pharmacological and therapeutic treatment of PBDs via the inhibition of the lysosome: block of pexophagy will most likely not universally be beneficial for all AAA complex mutants, but only for those proteins with mutations that cause milder defects and thus still allow the formation of a partially active protein.

In summary, our results on yeast Pex1 and the published original data on mammalian Pex1 can be combined to one general working model: Pex1 is directly and essentially involved in matrix protein import. *PEX1* deletion-induced pexophagy might possibly be the response to the matrix protein import defect, but it is clearly not responsible for it. The peroxisomal PTS1 import defect in *PEX1*-deficient cells is independent of pexophagy.

## 4. Materials and Methods

### 4.1. Yeast Strains and Culture Conditions

The *Saccharomyces cerevisiae* wild-type strain UTL-7A (MATa, ura3-52, trp1, leu2-3/112) was used for the generation of the *pex1*Δ strain [24]. The wild-type strain BY4742 (MATα, *his3*Δ*1*, *leu2*Δ*0*, *lys2*Δ*0*, *ura3*Δ*0*) and the *pex1*Δ mutant as well as the wild-type strain BY4741 (MATa, *his3*Δ*1*, *leu2*Δ*0*, *met15*Δ*0*, *ura3*Δ*0*) and the single deletion mutant *pex1*Δ were purchased from EUROSCARF (Frankfurt a.M., Germany) [25]. The double deletion mutant BY4741 *pex1*Δ*atg36*Δ was a kind gift from Ewald Hettema (Sheffield, UK) [13]. The complete (YPD), minimal media (SD) as well as oleic acid yeast medium (YNO) for inoculation and plates have been described previously [26]. 

### 4.2. Plasmids

The plasmids Pex11-GFP [27] and GFP-PTS1 [28] were described previously. 

### 4.3. Fluorescence Microscopy

Analysis of live cells for green fluorescence protein (GFP) fluorescence was performed with a Zeiss Axioplan microscope and AxioVision 4.1 software (Zeiss, Jena, Germany) as described before [29]. FM4-64 (T3166) was purchased from Invitrogen (Karlsruhe, Germany).

### 4.4. Pexophagy Assay

For the pexophagy assay based on [30], yeast strains expressing the peroxisomal membrane protein Pex11 C-terminally fused with GFP were grown in two precultures (20 mL overnight and 50 mL for 8 h, OD_600nm_ = 0.3) in SD medium at 30 °C. Peroxisomal proliferation was induced by incubating the cells for 16 h at 30 °C (OD_600nm_ = 0.5) in 100 mL oleate media. To induce pexophagy, the cells first had to be harvested at 4000 rpm for 5 min at 4 °C and washed two times with 5 mL sterile dH_2_O (5 min, 4000 rpm, 4 °C). Cells were resuspended in 1 mL sterile water, and 0.5 mL of cell suspension was transferred to 100 mL nitrogen starvation media (SD(-N)). Samples of the starting point (T0 samples, 0.5 mL remaining of cell suspension) were taken immediately, harvested for 5 min at 4000 rpm and prepared by TCA precipitation. The culture was incubated for 23 h at 30 °C. After 23 h, the T23 samples (50 mL) were harvested, washed two times and as well prepared by TCA (trichloroacetic acid) precipitation (as described in [24]).

### 4.5. Yeast Cell Fractionation

The spheroplasting of yeast cells, their homogenization and the subsequent differential centrifugation at 25,000× *g* of post-nuclear supernatants were performed as described previously [26].

### 4.6. Immunodetection

Polyclonal rabbit antibodies were raised against Pex13 [31] and Por1 [32]. Monoclonal mouse antibodies were raised against GFP (Sigma-Aldrich/Merck, Germany) and Pgk1 (Invitrogen, Karlsruhe, Germany). Immuno-reactive complexes were visualized using the IRDye 800CW goat anti-rabbit IgG or IRDye 680RD goat anti-rabbit secondary antibody (Li-COR Bioscience, Bad Homburg, Germany) followed by detection using the “Infrarot Imaging System“ (Li-COR Bioscience, Bad Homburg, Germany). The intensity of free anti-Pex13 signals on the Western blots was calculated by Image Studio Lite, LI-COR Bioscience.

### 4.7. Growth on Liquid Oleate Medium

The functionality of peroxisomes was monitored by measuring the OD600 of the cells grown in YNO. Cells were first precultured for 16 h in 25 mL SD medium and transferred to 500 mL YNO with a starting OD600 of 0.1. OD600 was measured after 42 h (*n* = 3), and data were corrected for the measured value of the negative control. The corrected data are displayed in % complementation activity compared to wild-type cells.

### 4.8. Statistical Analysis

The results of the experiments (*n* = 3) are presented as means ± standard deviation (SD). The analysis of variance was performed by use of *t*-test procedures. A *p*-value *p* < 0.001 (***) was considered as significant.

### 4.9. Nomenclature

In order to simplify the text and make the protein and genes names independent from the species, we use the following nomenclature in this manuscript: Pex1 (protein); *PEX1* (gene); *pex1*Δ or *PEX1*-deleted cells (strain/cells with completely deleted gene).

## Supplementary Materials

Supplementary materials can be found at https://www.mdpi.com/1422-0067/21/3/867/s1.
