# Supplementary material for: The Peroxisomal PTS1-Import Defect of PEX1- Deficient Cells Is Independent of Pexophagy in Saccharomyces cerevisiae
Source: Int J Mol Sci. 2020 Jan 29;21(3):867. doi: 10.3390/ijms21030867 (PMC7037794; doi:10.3390/ijms21030867)
Supplement: Supplementary file 1 [file ijms-21-00867-s001.pdf]

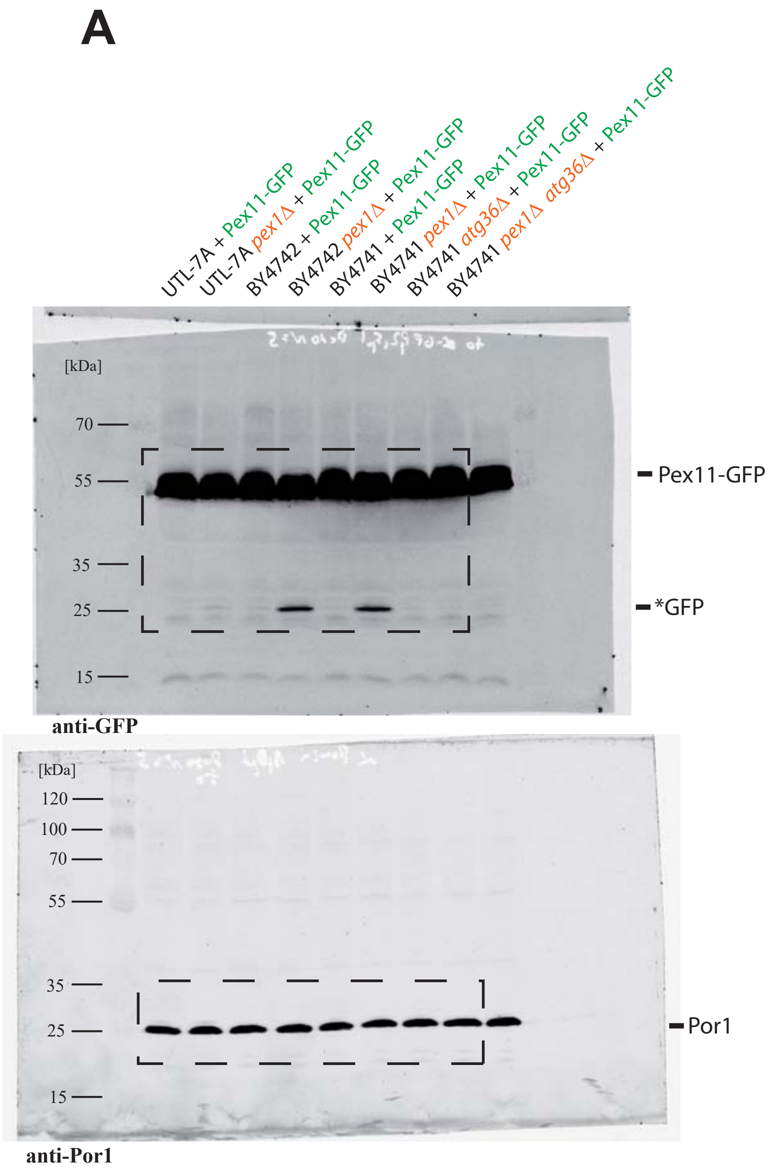

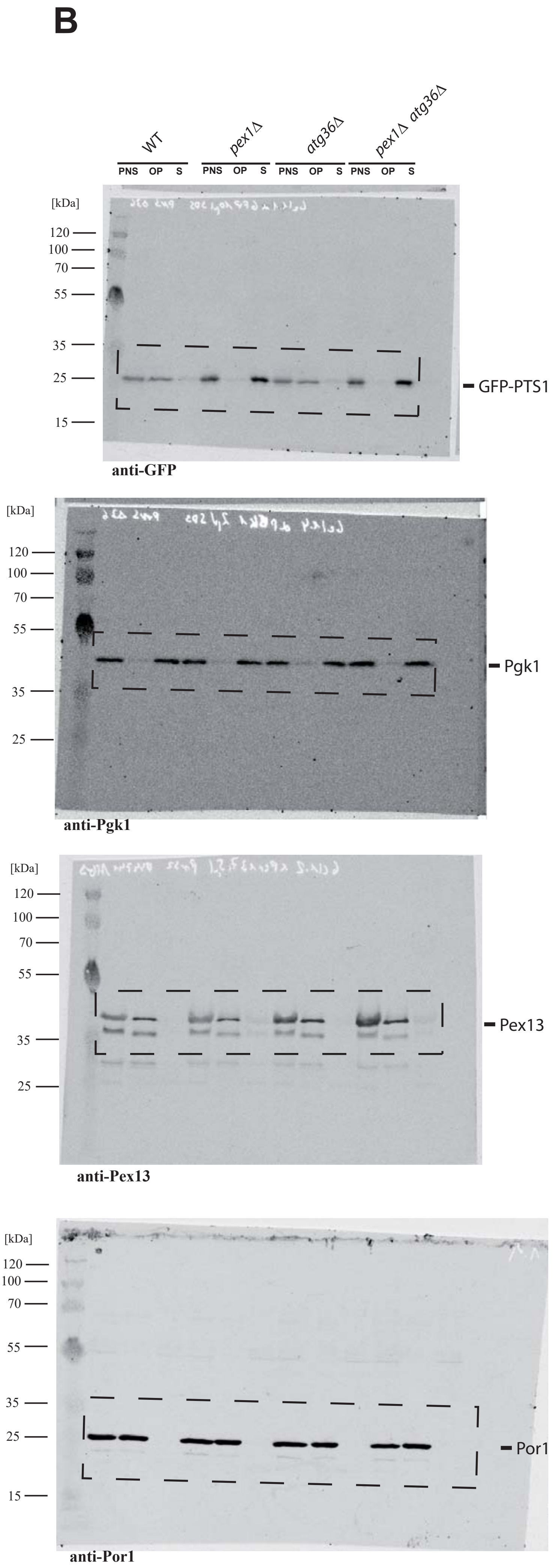

Supplementary Figure S1

(A) Uncropped Western Blots corresponding to Figure 1A (marked area). (B) Uncropped Western Blots corresponding to Figure 1D.
